# Supplementary material for: T Cell Responses in Pregnant Women Who Received mRNA-Based Vaccination to Prevent COVID-19 Revealed Unknown Exposure to the Natural Infection and Numerous SARS-CoV-2-Specific CD4- CD8- Double Negative T Cells and Regulatory T Cells
Source: Int J Mol Sci. 2024 Feb 7;25(4):2031. doi: 10.3390/ijms25042031 (PMC10889590; doi:10.3390/ijms25042031)
Supplement: Supplementary file 1 [file ijms-25-02031-s001.zip › ijms-2814733-supplementary.pdf]

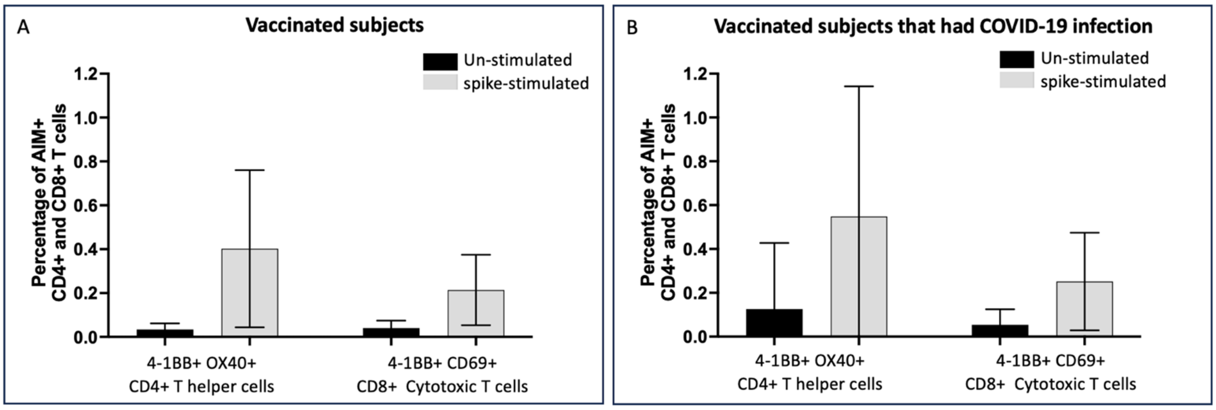

Supplementary Figure S1. Percentage of activated CD4+ and CD8T cells in the un-stimulated controls and spike-stimulated cultures. A. Vaccinated subjects that did not report COVID-19. B. Vaccinated subjects that had a previous COVID-19 infection.

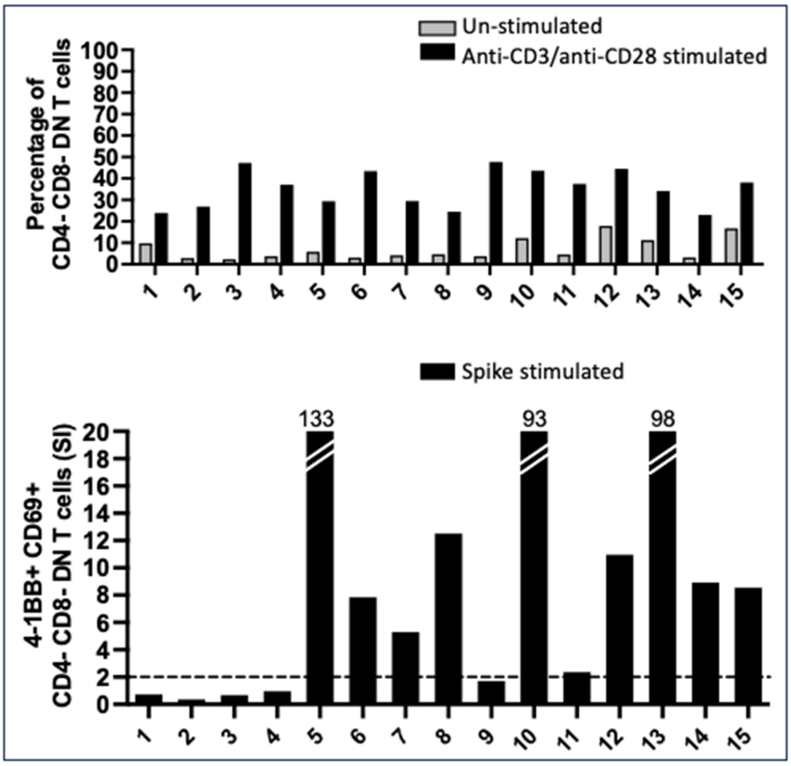

Supplementary Figure S2. DN T cells in healthy vaccine recipients. CD4-CD8- DN T cells enumerated in 14 female and male healthy recipients of mRNA-based vaccination for COVID-19 protection [13]. **A**. DN T cell responses to anti-CD3/CD28 stimulation. B. DN T cell responses in response to SARS-CoV-2 spike peptides, expressed as SI.
